# Renal Amyloidosis in Pediatric Autoinflammatory Syndromes: Subclinical Onset, Early Biomarkers, and Clues for Timely Characterization and Interventions

**DOI:** 10.3390/jcm15176795

**Published:** 2026-09-01

**Authors:** Naqiya Arsiwala, Donato Rigante

**Affiliations:** 1 Department of Life Sciences and Public Health, Fondazione Policlinico Universitario A. Gemelli IRCCS, I-00168 Rome, Italy; naqiyashabbir.arsiwala01@icatt.it; 2 Periodic Fevers Research Center, Università Cattolica Sacro Cuore, I-00168 Rome, Italy

**Keywords:** autoinflammatory syndromes, AA amyloidosis, familial Mediterranean fever, serum amyloid-A, urinary neutrophil gelatinase-associated lipocalin (NGAL), interleukin-1 inhibition, anakinra, canakinumab, subclinical inflammation, innovative biotechnologies, renal surveillance, pediatric nephrology, personalized medicine

## Abstract

**Background/Objectives:** Autoinflammatory syndromes are defined by episodes of recurrent fever driven by innate immune dysregulation, yet their long-term organ consequences remain largely underestimated, mostly in children. Renal AA amyloidosis represents the most perilous complication of sustained, even subclinical, inflammation, capable of progressing silently to irreversible renal failure. In pediatric cohorts, the interval between autoinflammatory disease onset and amyloidosis diagnosis spans nearly a decade, an actionable window that current surveillance frameworks have not adequately addressed. This review examines the pathophysiological *continuum* linking chronic cytokine overproduction to renal amyloid deposition in children with autoinflammatory syndromes, evaluates emerging subclinical biomarkers of early renal injury, identifies relevant diagnostic pitfalls, and proposes a structured renal surveillance framework for at-risk pediatric populations. **Materials and Methods:** This comprehensive review has been conducted analyzing studies reporting renal outcomes, biomarker data, or therapeutic interventions in pediatric patients with monogenic or polygenic autoinflammatory conditions. No date restriction was applied, with emphasis placed on publications from 2015 onward reflecting contemporary treatments and diagnostic standards. **Results:** An overproduction of interleukin (IL)-1β and IL-6 drives serum amyloid-A accumulation that may persist measurably during intercritical periods, preceding overt proteinuria by years in most young patients with autoinflammatory syndromes. Urinary neutrophil gelatinase-associated lipocalin (NGAL) is significantly elevated in attack-free children with familial Mediterranean fever (FMF) compared to healthy controls, representing an early and sensitive marker of tubular stress. Shear wave elastography has demonstrated measurable increases in renal tissue stiffness in pediatric FMF patients, correlating with subclinical inflammatory activity. Critically, nutcracker syndrome has emerged as the leading cause of proteinuria in colchicine-treated FMF cohorts, accounting for up to 67.5% of proteinuria cases and representing a clinically significant confounder. IL-1 inhibition with either anakinra or canakinumab has demonstrated histological regression of established amyloid deposits in different cohorts of pediatric patients, reinforcing the potential therapeutic value of earlier identification and intervention before overt irreversible glomerular damage comes out. **Conclusions:** Renal amyloidosis in autoinflammatory syndromes is a plausibly preventable outcome when subclinical injury is identified and treated within the pre-amyloid window in the pediatric age. Conventional reliance on ‘proteinuria’ as the primary surveillance threshold is potentially misleading. A biomarker-guided monitoring approach incorporating serum amyloid-A, urinary NGAL, and renal elastography might offer a clinically actionable pathway toward earlier nephrology referral and targeted IL-1 blockade, with the potential to improve renal prognosis of these children.

## 1. Introduction

Autoinflammatory syndromes occupy a clinical space defined by a deceptive paradox: episodes of fever and systemic inflammation recur with relentless predictability, and yet between attacks the affected child often appears entirely well [1,2]. This cyclical presentation has shaped clinical attention toward acute symptom management and attack prevention, leaving the slower cumulative consequences of inflammation comparatively underexamined. The recurrent fevers that bring these children to medical attention are, in the most consequential sense, a presenting feature rather than the disease itself. Among the long-term consequences of autoinflammatory flares, renal AA amyloidosis stands as the most clinically serious, resulting from persistent proinflammatory cytokine production, principally interleukin (IL)-1β and IL-6, which together drive hepatic synthesis of serum amyloid-A (SAA) protein [3]. When SAA remains chronically elevated, fragments of the protein misfold into amyloid fibrils that deposit preferentially within the glomerulus, but also in vessels and/or interstitium, producing a slowly progressive nephropathy that potentially culminates, if unchecked, in end-stage renal failure [3,4]. AA amyloidosis was historically the leading cause of mortality in patients with untreated familial Mediterranean fever (FMF), which is the prototypical and most-known hereditary autoinflammatory syndrome: although prophylaxis with colchicine has substantially reduced incidence of amyloidosis over the past four decades, the complication has not disappeared, particularly in patients with poor adherence to colchicine, insufficient or partial colchicine response, or limited access to colchicine use [5,6]. The median interval between diagnosis of an autoinflammatory syndrome and confirmed AA amyloidosis may span nearly a decade in children: that decade is not a passive interval, but a window during which low-grade glomerular and tubular injury accumulates silently, often producing detectable biochemical and structural changes long before overt proteinuria triggers nephrology referral [7,8]. Current surveillance practice, anchored largely in periodic urinalysis and assessment of clinical disease activity of the specific autoinflammatory syndrome, is based on the identification of amyloidosis once renal damage is already established rather than during the modifiable phase that precedes it.

**Figure 1:**
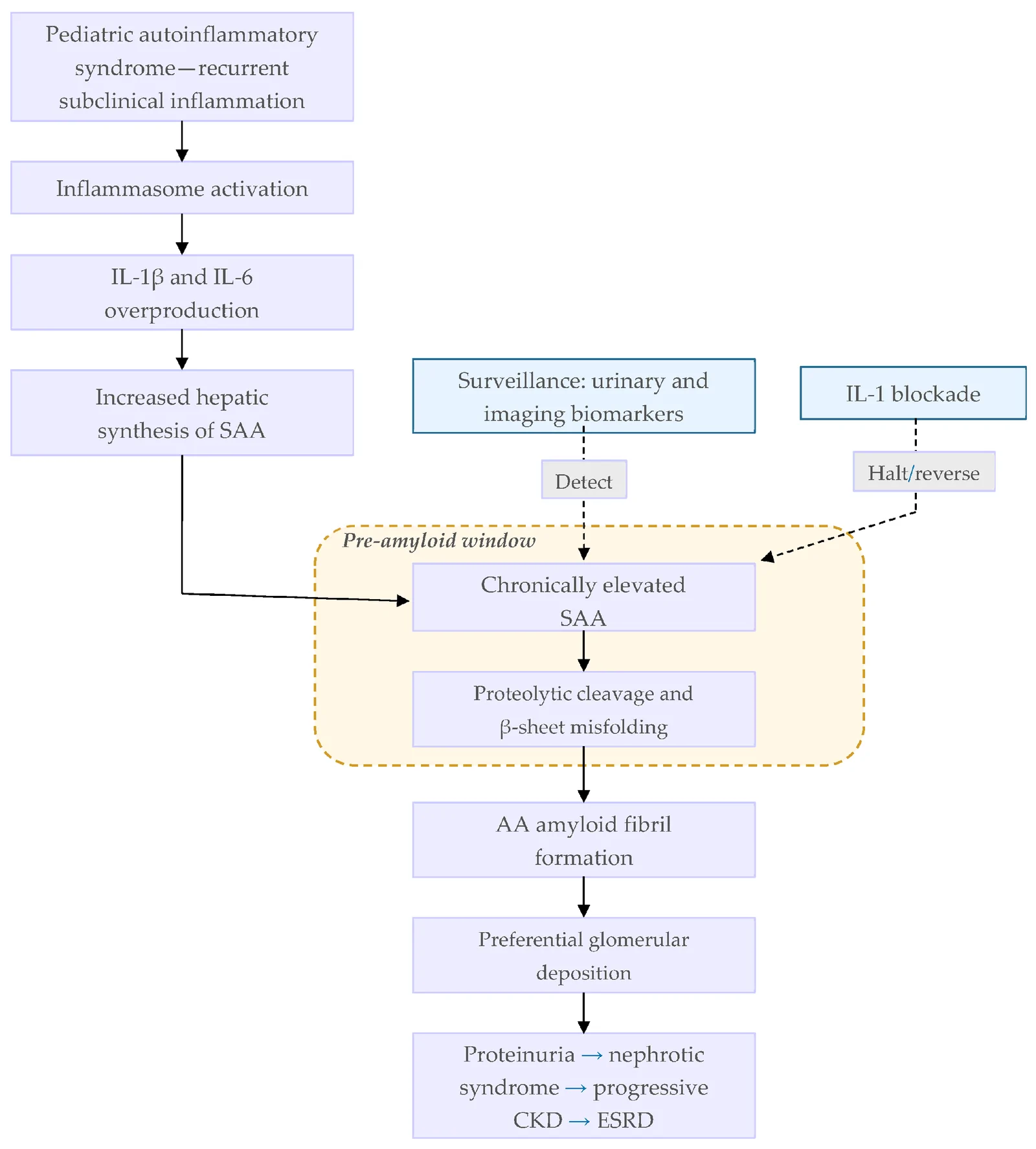
Pathogenic cascade from chronic inflammation to the onset of renal AA amyloidosis in autoinflammatory syndromes; the shaded band marks the *pre-amyloid window*, i.e., a feasibly modifiable phase responsive to biomarker-guided surveillance and interleukin (IL)-1 blockade. *Abbreviations:* IL-1β, interleukin-1 beta; IL-6, interleukin-6; SAA, serum amyloid-A; CKD, chronic kidney disease; ESRD, end-stage renal disease.

This review argues that the pre-amyloid window may be detectable, characterizable, and clinically actionable (see Figure 1). By integrating evidence on subclinical inflammatory persistence, emerging urinary and imaging biomarkers, and the documented capacity of cytokine-targeted therapies to halt and in selected cases, possibly reverse amyloid progression, this work proposes a structured renal surveillance framework for children with autoinflammatory syndromes. Building on the conceptual foundation that autoinflammatory syndromes are systemic diseases whose impact extends well beyond fever phenotype, the focus here is specifically on what happens to the kidney during the years before clinicians traditionally start looking and on the practical question of how that period can be made visible to routine pediatric care.

## 2. Materials and Methods

This narrative review summarizes the current medical literature on renal involvement in the autoinflammatory syndromes of childhood, with particular attention to subclinical injury, emerging biomarkers, and therapeutic implications relevant to AA amyloidosis. Literature searches were performed in PubMed, which indexes MEDLINE, and Scopus, from database inception to July 2026, the date of the last search. Search terms combined the autoinflammatory conditions of interest with terms for renal involvement, candidate biomarkers, and treatment. A representative string, adapted to each database, was (“familial Mediterranean fever (FMF)” OR “tumor necrosis factor receptor-associated periodic syndrome (TRAPS)” OR “cryopyrin-associated periodic syndrome (CAPS)” OR “systemic juvenile idiopathic arthritis (sJIA)” OR “mevalonate kinase deficiency” OR “autoinflammatory”) AND (“amyloidosis” OR “renal involvement” OR “proteinuria” OR “microalbuminuria” OR “neutrophil gelatinase-associated lipocalin (NGAL)” OR “serum amyloid-A” OR “shear wave elastography (SWE)” OR “colchicine” OR “anakinra” OR “canakinumab” OR “interleukin-1 inhibition” OR “tocilizumab”). Reference lists of key-articles were screened manually for additional sources. Records were restricted to English-language publications. Studies were included if they reported renal outcomes, biomarker performance, or therapeutic responses in patients with monogenic or polygenic autoinflammatory syndromes, with priority given to pediatric data; original research, multi-centre cohorts, registry analyses, clinical guidelines, and systematic reviews were given precedence. Studies were excluded if they did not address these domains, or if pediatric data could not be distinguished within predominantly adult cohorts, unless the findings were foundational. Emphasis was placed on publications from 2015 onward, with earlier work retained where it remains a definitive reference, including the establishment of colchicine in FMF and the natural-history studies of AA amyloidosis. Screening and selection were performed by the first author under the supervision of the senior author.

Because the evidence spans mechanistic, biomarker, and therapeutic domains of differing methodological strength, sources were appraised qualitatively rather than pooled. Prospective, multi-centre, registry, and guideline-level evidence was weighted most heavily; findings from small, single-centre, or cross-sectional studies, including much of the current pediatric evidence on urinary NGAL and renal SWE, were treated as hypothesis-generating and are identified as such throughout. This appraisal is reflected in the strength-of-evidence descriptors accompanying the surveillance framework. Consistent with a narrative design, this work does not constitute a systematic review, and a formal protocol with quantitative record accounting and a PRISMA flow diagram were not undertaken.

## 3. The Renal Vulnerability of Autoinflammatory Syndromes

### 3.1. The Spectrum of Autoinflammation with Risk of Renal Involvement

The clinical landscape of pediatric autoinflammatory syndromes has expanded considerably over the past two decades [1,2], yet the renal complications encountered in everyday practice remain concentrated within a narrow group of conditions [3,4]. Among hereditary monogenic autoinflammatory syndromes [9], three are responsible for nearly all clinically significant renal involvement seen in children, FMF, TRAPS, and CAPS, this last comprising familial cold-induced autoinflammatory syndrome, Muckle–Wells syndrome, and neonatal-onset multisystem inflammatory disease (NOMID), while sJIA, currently renamed Still’s disease as its adult counterpart, is increasingly grouped within the autoinflammatory spectrum [1,2,9]. Each of these conditions reaches the kidney through a shared final pathway of sustained proinflammatory cytokine excess, though differing meaningfully in the timing, severity, and predictability of this journey [4]. FMF carries the heaviest historical burden of AA amyloidosis [5,10]: caused by variants in *MEFV*, which encodes the pyrin protein, this disease has been associated with amyloidosis prevalence exceeding 60% in untreated cohorts of patients living around the Eastern Mediterranean sea [5], a figure now largely of historical interest in colchicine-treated populations but still relevant in patients with poor adherence or limited access to treatment protocols [6,11,12]. Genotype contributes meaningfully to risk stratification [10,13], with homozygous M694V *MEFV* mutations conferring the highest amyloidogenic potential, while compound heterozygous and milder variants may display a mitigated risk [10,13]. A disease genetic confirmation in these patients therefore serves a dual purpose, informing not only the diagnosis itself but the intensity of surveillance and the threshold for therapeutic escalation that follows [6,10].

TRAPS, driven by mutations in *TNFRSF1A*, involving the biosynthesis of the type 1 receptor of tumor necrosis factor, follows a different and arguably more deceptive course. Up to a quarter of untreated patients may develop renal amyloidosis, and this can occur even when the systemic disease activity appears clinically mild [4,14]. This mismatch between outward calm and internal progression makes TRAPS demanding to monitor, since a child who appears well to family and clinician alike may already be accumulating renal injury that will declare itself only years later [7,14]. Tumor necrosis factor inhibitors were initially used to treat TRAPS, but their role is now limited, while IL-1 inhibitors like both anakinra and canakinumab are the preferred targeted first-line biologic therapies [15,16]. Surveillance frameworks calibrated to FMF, where attack frequency tracks reasonably well with biochemical activity, can underestimate TRAPS-related risk if applied without personalized approaches to the individual patient [14,17].

CAPS, caused by *gain-of-function* variants in *NLRP3*, encoding an intracellular sensor (named cryopyrin) that detects a broad range of microbial motifs, has historically carried a lower amyloid risk than FMF and TRAPS, but the most severe cases of NOMID are capable of producing amyloidosis if inadequately controlled with IL-1 inhibitors [18,19,20]. The introduction of anakinra and canakinumab into CAPS management has yielded one of the cleanest natural experiments in modern rheumatology, demonstrating that sustained IL-1 blockade can modify the clinical natural history and arrest amyloid progression in this population [20,21]. IL-1 blockade has been successful even in the most complicated pictures of CAPS involving the central nervous system [22]. This evidence is foundational to the broader argument advanced throughout this review: that early cytokine-targeted therapy alters long-term renal outcome rather than simply managing symptoms [7,21].

sJIA occupies a more complex territory, as the reported amyloidosis incidence varies between 1 and 10% across geographic regions and treatment eras [22], a range that reflects both genuine epidemiological heterogeneity and changing therapeutic landscape over the past two decades [23]. Beyond classical amyloid pathways, sJIA carries an additional hazard of macrophage activation syndrome, which may precipitate acute kidney injury through mechanisms entirely distinct from chronic amyloid deposition [24,25]. Renal risk in this population must therefore be assessed along a personalized surveillance approach which is not the same for monogenic autoinflammatory syndromes.

### 3.2. Why the Kidney Bears the Brunt of Amyloidosis

If chronic inflammation is the engine of AA amyloidosis, the renal glomerulus is the district structurally predisposed to receive its output, as shown by different studies related to FMF [3,4,25]. Its high filtration surface area, fenestrated endothelium, and continuous exposure to plasma SAA conspire to make it the predominant site of AA fibril deposition in systemic amyloidosis [3]. Deposition typically begins within the mesangium [3,25] and progresses outward over time to involve capillary walls, basement membranes, and ultimately the tubule-interstitial compartment [3]. The resulting nephropathy follows a recognizable trajectory: silent deposition gives way to microalbuminuria, then to overt proteinuria and nephrotic syndrome, leading to irretractable damage with declining glomerular filtration, and finally to end-stage disease [3,4,25]. Childhood vulnerability to this sequence is not simply a matter of body size or organ scale: the pediatric kidney faces a temporal asymmetry that the adult kidney does not, because a child diagnosed with FMF at three years of age and inadequately controlled has potentially decades of subclinical amyloidogenic stress ahead before reaching adulthood [7,25]. The window for primary prevention is therefore wider in children than in any adult cohort, and the consequences of failing to intervene within it are correspondingly more severe [7]. This observation reframes the clinical task: the question in pediatric autoinflammatory syndromes is not when amyloidosis will declare itself, but whether it can be prevented from declaring itself at all [7,21]. A note on terminology is warranted. That biomarkers rise before overt proteinuria shows subclinical renal stress is detectable early, but it does not establish that this period precedes amyloid deposition or that the injury is still reversible, since rising biomarkers may reflect deposition already underway below the threshold of detection. We therefore use “pre-amyloid window” as an operational construct, defined by biomarker positivity without proteinuria, rather than a histologically verified amyloid-free state, and treat it as a testable hypothesis about timing that paired histological and longitudinal studies will need to confirm.

### 3.3. The Mechanism of Subclinical Persistence of Inflammation

**Figure 2:**
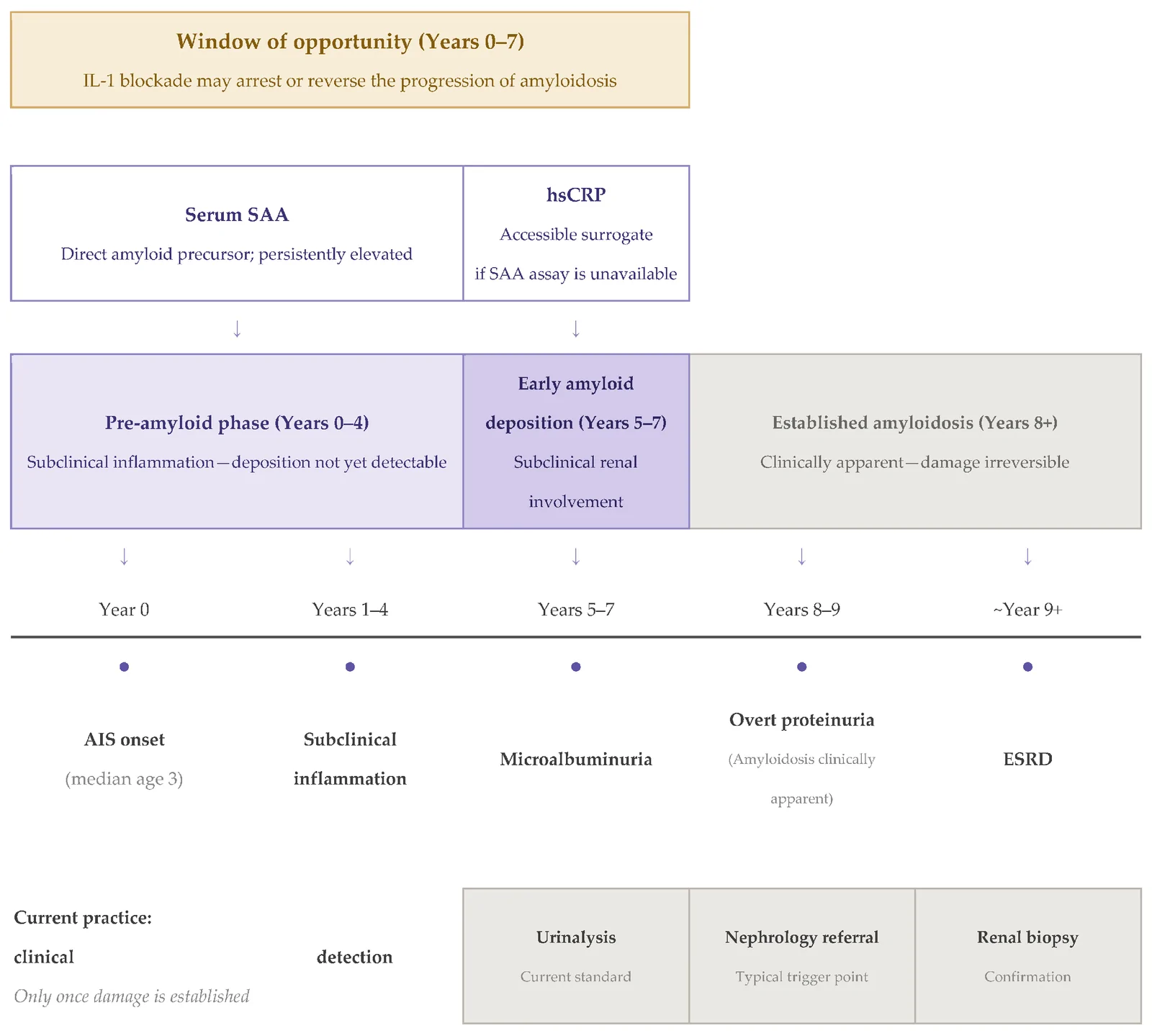
Natural history of renal involvement in autoinflammatory syndromes, exemplified by the model of familial Mediterranean fever (FMF). Intercritical elevation of serum amyloid-A and C-reactive protein is detectable years before the first clinically actionable renal abnormality; under current practice, detection is disclosed only at microalbuminuria, by which point glomerular amyloid deposition may already be established. The proposed strategy shifts detection into the pre-amyloid phase, where interleukin-1 blockade may potentially arrest progression. The timeline is FMF-derived and illustrative; its sequence and duration vary across the other syndromes, and the “pre-amyloid” phase is defined operationally by biomarker positivity before overt proteinuria, its relationship to actual amyloid deposition awaiting histological confirmation. *Abbreviations:* AIS, autoinflammatory syndrome; IL-1, interleukin-1; SAA, serum amyloid-A; hsCRP, high-sensitivity C-reactive protein; ESRD, end-stage renal disease.

The single most important conceptual shift in understanding autoinflammation-related renal injury has been the recognition that these chronic diseases are never ‘truly’ quiet between attacks [15,26]. Serial measurement of acute phase reactants, including SAA and high-sensitivity C-reactive protein (hsCRP), has demonstrated that their levels may remain elevated during intercritical periods in a substantial proportion of patients judged clinically well by their treating physicians [17,27,28]. This persistent low-grade inflammation as a low heat maintains a continuous amyloidogenic substrate that standard disease activity scores were never designed to detect, and the implication for clinical practice is direct: the absence of fever does not mean absence of damage [17,26]. The framing of an autoinflammatory syndrome as an episodic disease, while convenient for both patient and clinician, has contributed to a systematic blind spot in pediatric care. If inflammation is continuous, then injury is continuous as well, and surveillance built around the visible markers of disease activity will inevitably miss the slower process operating underneath [17,26]. To fully grasp this hidden progression, it is essential to distinguish four biologically related but distinct states. First, persistent systemic inflammation acts as the underlying driver, characterized by chronically raised intercritical SAA that sustains the amyloidogenic substrate [15,26]. This progresses silently to subclinical renal injury, involving localized tubular epithelial stress and altered tissue compliance prior to any actual fibril deposition [28,29]. Next, early amyloid deposition begins primarily in the mesangium, functionally signaling itself through microalbuminuria, a phase where structural deposition is established but remains potentially reversible [3,25]. Finally, the disease reaches established renal AA amyloidosis, manifesting clinically with overt proteinuria, declining glomerular filtration and irreversible renal dysfunction [3,4]. Everything that follows in this review is, in essence, an attempt to make this continuum visible and actionable (see Figure 2).

## 4. Clinical Presentations of Renal Injury as a Continuum

The clinical manifestation of renal involvement in pediatric autoinflammatory syndromes unfolds along a *continuum* that traditional surveillance has compressed into a binary distinction of no proteinuria *versus* proteinuria [3,4]. This simplified binary obscures clinically meaningful intermediate stages during which intervention remains both possible and effective [7,21]. Recognizing the full *continuum* is therefore not an academic refinement but a precondition for changing outcomes, and the structure of this section follows the natural progression of injury rather than the artificial thresholds of conventional monitoring. The staged progression described here is best characterized in FMF and should not be assumed to apply uniformly across all pediatric autoinflammatory syndromes, which differ considerably in genetic mechanism, inflammatory phenotype, amyloid risk, treatment response, and pattern of renal involvement [1,4].

In FMF, the sequence typically unfolds over childhood, with a latency of several years between onset and clinically apparent amyloidosis [7,25]. In TRAPS, the interval is longer and more deceptive: amyloidosis affects a minority of patients, is concentrated in specific structural variants, and may declare itself only years later, at times when systemic disease has appeared clinically mild [4,14]. CAPS-associated amyloidosis, largely confined to the Muckle–Wells and NOMID phenotypes, has historically emerged later and has been substantially modified by sustained IL-1 blockade [18,20,21], while amyloidosis in mevalonate kinase deficiency is uncommon and, when it occurs, is frequently revealed by renal involvement itself [1]. sJIA occupies separate ground: beyond a variable and generally declining amyloid incidence, it carries a distinct risk of acute kidney injury through macrophage activation syndrome, a mechanism unrelated to chronic amyloid deposition [24].

The pre-amyloid window is therefore best understood not as a single fixed timeline but as a syndrome-dependent interval, longest and best defined in FMF and more variable, and at times more compressed or clinically silent, elsewhere. That its boundaries are not yet precisely defined does not diminish its clinical importance; on the contrary, it is the interval in which the difference between reversible and irreversible injury is likely decided, and defining it prospectively across the different syndromes is a central empirical task this review identifies. In the earliest stage, which often extends across several years, the affected child shows no urinary abnormality detectable on standard dipstick testing [25].

Clinical attention during this period is focused predominantly on attack frequency, fever control, and the symptomatic burden of recurrent inflammation [6,27]. Renal injury, however, is already underway at the molecular and microstructural level, with mesangial amyloid accumulation, tubular epithelial stress responses, and incremental changes in renal tissue compliance—all preceding any biochemical signal that routine practice is equipped to detect [3,25,28]. A normal urinalysis in a child with active or recently active autoinflammatory syndrome does not exclude renal involvement; it confirms only that conventional monitoring lacks the sensitivity to catch and register it [25,29]. An intermediate stage of renal involvement is defined by the convergence of three phenomena that together represent the strongest single argument for changing how pediatric autoinflammatory syndromes should be monitored. First, microalbuminuria emerges as the earliest conventionally measurable sign of glomerular dysfunction, though by the time it appears, mesangial amyloid deposition could be already established [3,4]. Second, NGAL may be elevated in attack-free children with FMF compared with healthy controls, reflecting tubular stress that precedes glomerular failure in this specific condition [29]. Third, SAA may remain raised during intercritical periods in a substantial proportion of clinically well patients, sustaining the amyloidogenic substrate even when the disease appears controlled [15,26]. The shared significance of these findings is temporal: they identify a period during which renal injury may be detectable before it becomes structurally irreversible. Therapeutic intervention initiated within this window has, in pediatric series, demonstrated a brilliant opportunity for histological regression of amyloid deposits and stabilization of renal function [7,21]. In the late phase, overt proteinuria appears and typically progresses rapidly toward nephrotic-range protein loss, hypoalbuminemia, peripheral edema, and hyperlipidemia [3,4]. Estimated glomerular filtration rate begins to decline, often more steeply than the proteinuria trajectory alone would predict [3,4].

Renal biopsy at this stage may reveal extensive amyloid deposition involving mesangial, capillary wall, and tubule-interstitial structures, with established glomerulosclerosis of different severities [3]. End-stage disease, requiring renal replacement therapy or transplantation, marks the failure of all upstream opportunities for prevention [4,23]. Outcomes following progression to this phase remain poor, with five-year survival historically reduced even in patients receiving optimal supportive care [4,23]. The pediatric epidemiology of this trajectory frames the clinical task with unusual precision: in a series of children treated with anti-IL-1 agents for AA amyloidosis secondary to autoinflammatory syndromes, the median age at disease onset was three years, while amyloidosis was diagnosed at a median age of twelve years [7]. The nine-year interval between these two events is not a measure of how slowly the disease progresses, but a measure of how late current diagnostic thresholds detect it. The same interval, viewed from the opposite direction, represents the period during which biomarker-positive but symptom-negative children can plausibly be identified, monitored, and treated before irreversible damage occurs [7,17,29]. The *continuum* framing matters because it identifies, with reasonable specificity, both when and with what tools that opportunity can be acted upon.

## 5. Early Detection of Amyloidosis: Biomarkers and Emerging Tools

### 5.1. Limitations of Proteinuria as an Early Clue of Renal Amyloidosis

Routine dipstick urinalysis and assessment of the urinary albumin-to-creatinine ratio remain fundamental components of renal monitoring in most pediatric guidelines for autoinflammatory syndromes [6,27]. These tests are inexpensive, widely available, and easy to implement, explaining their continued incorporation into routine surveillance protocols. However, an important limitation is that they generally identify renal involvement only after a structural damage has already occurred [3,25]. By the time persistent proteinuria becomes detectable, glomerular amyloid deposition is often well established, and a substantial proportion of nephrons may already have sustained irreversible injury [3,4]. Consequently, surveillance strategies that rely primarily on proteinuria may fail to identify patients during the earliest stages of renal amyloid deposition, when intervention is most likely to plausibly preserve kidney function [26,29].

### 5.2. Biochemical Markers of Subclinical Renal Injury

Three biochemical markers have emerged as candidates for moving renal surveillance upstream of overt proteinuria: urinary NGAL, microalbuminuria, and SAA, as shown by Table 1. Each of these measures a different stage of the injury *continuum*, and their combined interpretation is more informative than any single value considered alone [15,26,29]. NGAL is a small protein expressed by tubular epithelial cells in response to ischaemic or inflammatory, and toxic injuries [30,31]. It also participates in broader inflammatory and tissue-remodeling processes, notably through its association with matrix metalloproteinase-9 [32]. This wider biology cuts two ways: it underlies NGAL’s sensitivity to early renal stress but also means its elevation is not specific to amyloidosis, and may reflect several inflammatory or tissue-injury pathways. This reinforces that NGAL is best used within a multimodal panel rather than as an isolated marker, and that disease-specific prospective studies are needed to establish whether it can identify children who will progress to renal AA amyloidosis. Its release into urine is rapid and largely independent of glomerular filtration, which makes it well suited to detect tubular involvement before glomerular markers shift [30,31]. In detail, urinary NGAL is significantly elevated in attack-free children with FMF compared with age-matched healthy controls, indicating persistent tubular stress even when patients are clinically asymptomatic [28]. Different FMF-related studies have reported correlations between urinary NGAL levels and disease severity scores, *MEFV* genotype, and new-onset proteinuria, supporting its role not only as a screening tool but as a marker of cumulative renal stress [29,30]. However, the main limitation of NGAL at present is the absence of validated cutoff values in the pediatric population, specific for the different autoinflammatory syndromes, with most data deriving from comparisons among healthy controls rather than from prospective studies tracking which thresholds predict subsequent amyloidosis [29,30].

It is important to distinguish between a biomarker that reflects subclinical renal stress or injury and one that has demonstrated prospective predictive validity for AA amyloidosis. On current evidence, urinary NGAL and renal SWE belong to the former category: both are elevated in children with FMF relative to healthy controls and are biologically plausible markers of tubular stress and altered renal compliance, but neither has been shown, in prospective study, to predict subsequent amyloidosis development [28,29]. They should therefore be regarded as markers of association and biological plausibility rather than validated predictors, a distinction that constrains the weight they can bear in surveillance decisions.

Microalbuminuria, conventionally defined as urinary albumin excretion between 30 and 300 mg per day, has long served as a screening marker for early glomerular dysfunction and has been adopted into FMF surveillance protocols by many centres [6,27]. Its limitation in the context of all autoinflammatory syndromes is one of timing rather than accuracy [3,25]. By the time microalbuminuria appears, mesangial amyloid deposition is typically already established, and the patient has moved out of the truly subclinical phase that surveillance is intended to capture [3,25]. Microalbuminuria is therefore better understood as a confirmatory rather than an early warning marker, and its negativity should not provide reassurance in the presence of elevated NGAL or persistently raised SAA [17,29].

SAA occupies a unique dual role in autoinflammation: it is the precursor protein from which AA fibrils are derived, and is simultaneously a sensitive acute phase reactant whose plasma concentration reflects ongoing inflammation [3,17]. A persistent elevation of SAA during intercritical periods of most autoinflammatory syndromes is one of the strongest predictors of amyloidosis development [17,26]. The implication for surveillance is direct: SAA monitoring should not be reserved for periods of active flare but incorporated into routine assessment, with sustained intercritical elevation serving as a clue for treatment escalation rather than continued observation [17,26]. The challenge with SAA is related to its accessibility, since quantitative SAA assays are not universally available in pediatric centres and lack standardized validated reference ranges [17]. hsCRP has also been used as a surrogate in many settings, but its correlation with SAA is imperfect, and reliance on C-reactive protein (CRP) alone might underestimate the true amyloidogenic risk in patients with discordance between SAA and CRP [17,26].

### 5.3. Structural and Histological Assessment of the Kidney in Autoinflammatory Syndromes

Where biochemical markers report on functional injury, imaging and histological tools should be used to assess the structural consequences of accumulating amyloid burden. Two modalities are relevant for pediatric surveillance: SWE as a non-invasive imaging technique, and renal biopsy as the definitive diagnostic standard. SWE provides a non-invasive ultrasonographic measurement of tissue stiffness, with applications now extending well beyond hepatology into renal assessment [28,33]. Specifically, SWE has demonstrated measurable increases in the renal cortical stiffness of children with FMF compared with healthy controls, with stiffness values correlating with overall disease activity, intercritical inflammatory markers, and proteinuria if present [28]. The proposed mechanism involves amyloid deposition, interstitial fibrosis, and microvascular changes contributing collectively to altered renal compliance, although the precise contribution of each remains to be quantified [28,33]. The appeal of SWE in pediatric surveillance lies in its non-invasive nature, absence of ionizing radiation, and its suitability for repeated longitudinal assessment [28,33]. The limitation, as with NGAL, relies in the absence of standardized acquisition protocols and validated reference data for the different autoinflammatory syndromes [28]. Nevertheless, most available studies support SWE as a complementary surveillance instrument in centres with appropriate ultrasound expertise, particularly where it can be integrated with biomarker findings to build a multi-modal assessment of renal status [28,29]. Conversely, renal biopsy with Congo red staining and immunohistochemistry for SAA is the ultimate diagnostic gold standard for AA amyloidosis [3,4]. Its significance is threefold: it confirms the presence and type of amyloid, distinguishing AA from other amyloid and non-amyloid glomerular disease; it establishes the extent and distribution of deposition, which carries prognostic weight; and it resolves diagnostic ambiguity when biomarker, imaging, and clinical findings conflict. However, it is an inappropriate screening tool, since the procedural risks, including bleeding and need for sedation in younger children, place it firmly within the diagnostic confirmation rather than surveillance category [3,4]. A surveillance framework for pediatric autoinflammatory syndromes must therefore be designed around non-invasive markers, with biopsy reserved to cases where biomarker abnormalities, clinical findings, and imaging coalesce to demand a definite histological clarification.

### 5.4. Towards an Integrated Biomarker Panel to Disclose Renal Amyloidosis

**Figure 3:**
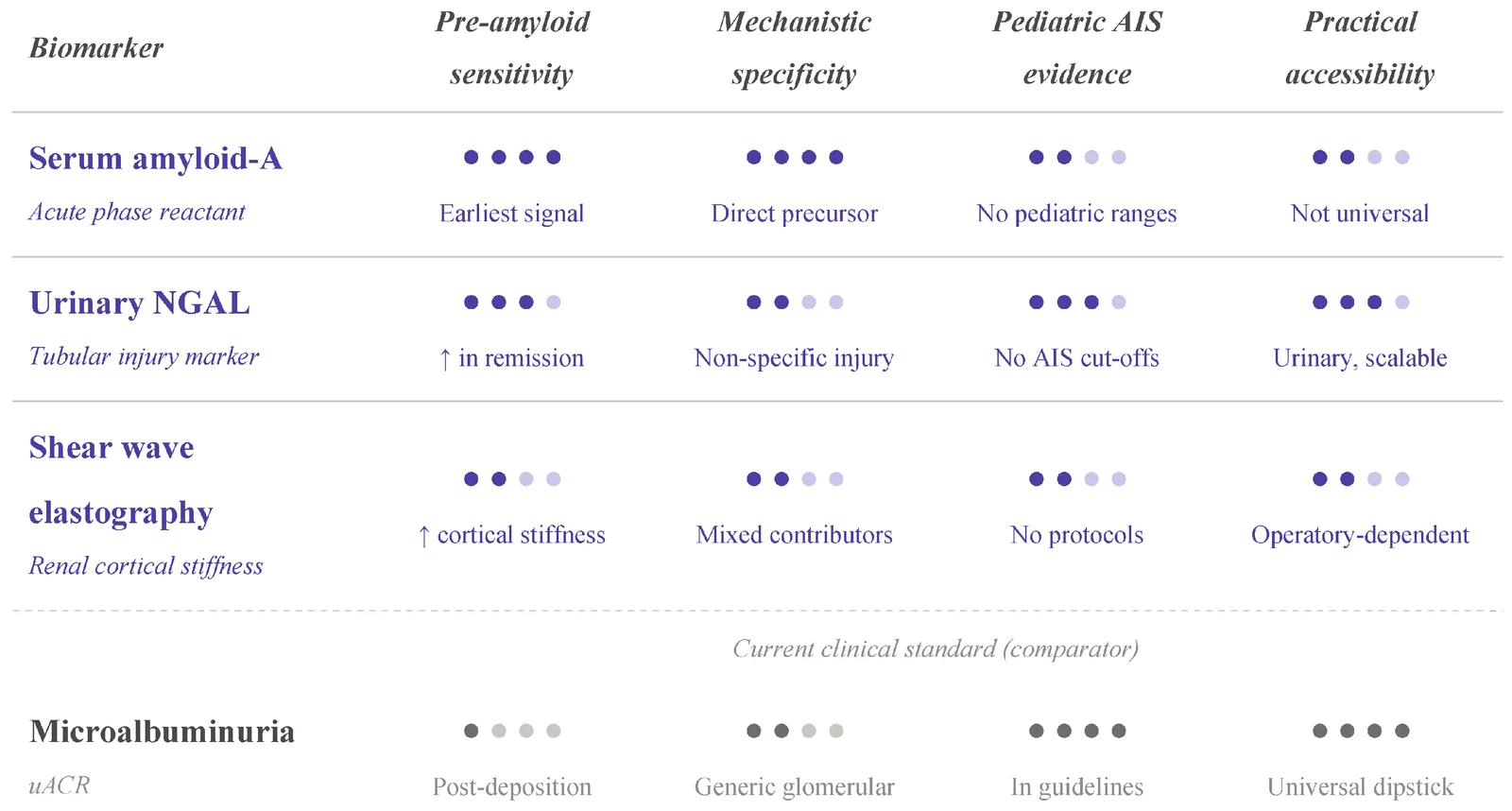
Qualitative comparison of candidate biomarkers (purple) and the current standard marker, microalbuminuria (grey), for the early detection of renal AA amyloidosis in pediatric autoinflammatory syndromes. Biomarkers are compared across four clinically relevant domains: sensitivity before overt amyloid-associated kidney injury (“pre-amyloid sensitivity”), mechanistic specificity, evidence in pediatric autoinflammatory syndromes, and practical accessibility. *Abbreviations:* AIS, autoinflammatory syndrome; NGAL, neutrophil gelatinase-associated lipocalin; SAA, serum amyloid-A; SWE, shear wave elastography; uACR, urinary albumin-to-creatinine ratio.

No single biomarker, on current evidence, possesses both the sensitivity and specificity required to drive surveillance decisions independently [17,29,30]. The most defensible approach, and the one this review proposes, is a layered panel combining urinary NGAL, SAA, urinary albumin-to-creatinine ratio, and renal SWE, applied at intervals appropriate to the syndrome-specific risk and general disease activity [6,17,28,29]. The diagnostic value of the panel lies in its concordance: discordant findings can be re-evaluated at shorter intervals, while concordant abnormalities across multiple modalities should trigger nephrology involvement and consideration of treatment escalation [7,21,29]. This integrated approach reframes surveillance programs in autoinflammatory syndromes from the detection of established disease to the characterization of an accumulating risk (see Figure 3, in which a qualitative comparison of candidate biomarkers and the current standard marker, microalbuminuria, is depicted for the early detection of renal AA amyloidosis across four domains: sensitivity before overt amyloid-associated kidney injury, mechanistic specificity, evidence in pediatric autoinflammatory syndromes, and practical accessibility).

## 6. The Confounder Position of the Nutcracker Syndrome

A surveillance framework reliant on proteinuria as the operative threshold faces a drawback that has recently received attention for FMF. In a recent pediatric cohort of 576 FMF children followed across ten years, the ‘nutcracker syndrome’—defined by compression of the left renal vein between the aorta and superior mesenteric artery—was identified as the leading cause of proteinuria, accounting for 67.5% of cases, while AA amyloidosis was responsible for only 15%, with no new amyloidosis diagnoses recorded over the previous eight years [35]. This shift in the epidemiology of proteinuria within colchicine-treated FMF populations represents both an indicator of therapeutic success and a new diagnostic challenge that contemporary surveillance frameworks have not fully absorbed [6,35].

This finding has two implications, neither of which is fully reflected in current clinical practice: the first is genuinely encouraging as in adequately treated FMF pediatric patients AA amyloidosis has become uncommon, reflecting the long-term effectiveness of standard colchicine prophylaxis [6,12,35]; the second is more cautionary, as the dominant cause of new-onset proteinuria in these patients is a structural vascular phenomenon unrelated to inflammation, and clinicians who reflexively attribute proteinuria to amyloidosis risk missing the true diagnosis [35]. The reverse error is equally consequential, since dismissing proteinuria as *nutcracker*-related without appropriate exclusion of amyloidosis can result in delayed treatment of amyloid disease in a minority of cases where it is genuinely present [3,35]. The clinical differentiation between these causes of proteinuria rests on a combination of imaging and biomarker assessment. Doppler ultrasonography of the left renal vein, including measurement of peak velocity ratios across the compression point, provides the principal diagnostic tool for the nutcracker syndrome, with computed tomography or magnetic resonance angiography reserved for ambiguous cases [35].

Children with nutcracker syndrome typically demonstrate lower body mass index, less subclinical inflammation, higher hemoglobin concentrations, and milder proteinuria with preserved serum albumin and estimated glomerular filtration rate compared with patients whose proteinuria has other causes [35]. Concurrently, the biomarker panel proposed earlier can help discriminate inflammation-driven renal injury from purely mechanical proteinuria, since elevated NGAL and persistently raised SAA point toward an inflammatory rather than a vascular etiology [29,31]. Where diagnostic doubt persists, a renal biopsy provides a definitive response [3].

A broader training is that to monitor renal function in children with autoinflammatory syndromes has become more diagnostically complex than current guidelines acknowledge. A monitoring framework cannot rely on any single test, because the same clinical finding, proteinuria, might signal either the successful prevention of one disease or the missed diagnosis of another [6,35]. This complexity is the inevitable consequence of therapeutic success, since reducing the incidence of one cause of proteinuria has, paradoxically, made the remaining causes harder to assess without a structured diagnostic approach [26,35]. The treatments examined in the next section operate against this evolving diagnostic backdrop. Their effectiveness depends not only on their pharmacological action, but on the surveillance infrastructure that identifies the right patients at the right time.

## 7. Treatment and the Pre-Amyloid Window

### 7.1. Role and Limitations of Colchicine in Amyloidosis Prevention

Colchicine has occupied the core of FMF management for more than five decades, and its impact on the natural history of disease remains one of the clearest success stories in pediatric rheumatology [5,6,12]. Colchicine, a RhoA (Ras homolog family member A) activator, stabilizes the pyrin inflammasome in FMF patients through a specific phosphorylation of pyrin [36]. Long-term adherence to colchicine administration reduces the attack frequency of FMF, suppresses subclinical inflammation, and substantially lowers the cumulative incidence of AA amyloidosis [13]. In adequately treated FMF cohorts, the annual incidence of new amyloidosis cases has fallen to near-zero in many centres, a reduction that explains the changing epidemiology of renal involvement discussed in the previous section [6,35]. Colchicine is not, however, universally protective.

Approximately 5–10% of FMF patients show partial or complete colchicine resistance, defined by the persistence of febrile attacks or by sustained elevation of acute phase reactants despite adequate colchicine dosing [6,37]. Specifically, these patients carry a residual amyloidosis risk that colchicine alone cannot address. Equally important, colchicine is not effective for TRAPS, CAPS and sJIA, in which the risk must be managed through different pharmacological pathways [14,20,21]. Colchicine and the principal pharmacological therapies related to renal AA amyloidosis in pediatric autoinflammatory syndromes are listed in Table 2.

### 7.2. IL-1 Blockade and Evidence for Amyloidosis Regression

The introduction of the recombinant IL-1 receptor antagonist anakinra and the long-acting anti-IL-1β monoclonal antibody canakinumab has fundamentally changed the therapeutic landscape for many hereditary causes of recurrent fevers [20,21]. In CAPS, canakinumab produces a dramatic suppression of clinical disease activity and normalization of acute phase reactants, as all CAPS manifestations are the product of IL-1 oversecretion [20]. De Benedetti et al. demonstrated the considerable efficacy of canakinumab in colchicine-resistant FMF, TRAPS, and mevalonate kinase deficiency, establishing IL-1 blockade as the principal second-line option across these autoinflammatory conditions [21]. The pediatric evidence on amyloidosis is more limited, but multi-centre series have also reported sustained reduction in attack frequency and biochemical markers of inflammation across mixed pediatric and adult colchicine-resistant cohorts of FMF patients treated with either anakinra or canakinumab [37]. Furthermore, starting from 65 children with colchicine-resistant FMF (with colchicine used at the maximum tolerable dose), all treated with canakinumab for at least one year given subcutaneously every 4 weeks at the dose of 150 mg—if they weighed over 40 kg—a significant reduction of proteinuria and stabilization of renal function were observed in a subgroup of 4 patients out of 65, who had an amyloidosis-related nephrotic syndrome. These patients maintained canakinumab treatment without interruption: in detail, two of them had a significantly decreased proteinuria with colchicine and canakinumab given for three years, though proteinuria persisted within the nephrotic range; the other two patients with FMF-related amyloidosis fully abolished their proteinuria after three years of canakinumab combined with colchicine [38].

Similar outcomes including partial remission of nephrotic syndrome in children with FMF and biopsy-confirmed renal amyloidosis were also reported with anakinra [38]. Importantly, clinical and histological outcomes must be distinguished. While IL-1 blockade consistently suppresses systemic inflammation (lowering SAA levels) and stabilizes glomerular filtration rate, reductions in proteinuria do not automatically equate to immediate clearance of amyloid fibrils [3,4]. Nonetheless, in selected pediatric cohorts, such as the one reported by Papa et al. with TRAPS-associated amyloidosis, sustained anti-IL-1 therapy not only preserved renal function and reduced proteinuria but also achieved biopsy-confirmed histological regression of amyloid deposits [7]. However, it must be emphasized that the available evidence for such structural regression in children is currently based on small observational series and selected case reports [7,38]. In the absence of robust comparative trials, expectations regarding the true reversibility of established amyloid disease must remain cautiously balanced. These observations carry important implications, but the therapeutic question is no longer whether established amyloid disease can be modified: it is whether it can be prevented entirely if a clinician’s intervention occurs early.

### 7.3. IL-6 Inhibition and Evidence for Amyloidosis Regression

Tocilizumab, a humanized monoclonal antibody acting against the IL-6 receptor, has shown efficacy in sJIA and has been used in selected cases of refractory AA amyloidosis, with some patients displaying reduced levels of SAA and stabilization of renal function [39]. The mechanistic rationale is direct, since IL-6 is a major driver of hepatic SAA synthesis, and its blockade interrupts the upstream supply of amyloidogenic substrate [3,39]. This drug is particularly effective in sJIA-associated amyloidosis, where IL-6 contributes more substantially to disease pathophysiology than in the classic monogenic autoinflammatory syndromes [23,39]. The likelihood of reversing renal damage is inversely proportional to the duration and extent of established amyloid deposition at the time of intervention [7].

Patients treated in the pre-amyloid or early amyloid-deposition phase show the most favorable renal outcomes, while those treated only after nephrotic-range proteinuria or impaired glomerular filtration have developed retain a substantially higher risk of progression to end-stage renal disease [4,7]. This evidence is the foundation of the surveillance argument advanced throughout this review: biomarker-guided monitoring is valuable not as an end in itself, but because it identifies the moment at which treatment escalation has the greatest probability of altering long-term outcome.

## 8. Toward a Renal Surveillance Framework: A Proposal for Prospective Evaluation

The framework outlined in this section draws together the biomarker, treatment, and epidemiological evidence reviewed previously. It is offered explicitly as a hypothesis-generating proposal for prospective evaluation, not as a clinically validated protocol for immediate adoption, and every element below should be read in that light. It reflects three principles that are consistently supported across the medical literature: (a) detection should target the pre-amyloid window rather than wait for clinical thresholds, (b) monitoring intensity should be calibrated to the syndrome-specific risk, and (c) treatment escalation may benefit from biomarker-guided decisions in combination with clinically based choices [6,7,17,29,35].

### 8.1. Who and When

All children with a confirmed diagnosis of monogenic autoinflammatory syndrome, including FMF, TRAPS and CAPS, are candidates for structured renal surveillance from the time of diagnosis [6,25]. Patients with mevalonate kinase deficiency tend to develop AA amyloidosis less frequently compared to the other hereditary causes of recurrent fevers in childhood; however, a renal surveillance program must also be pursued in this peculiar subset of patients [1]. Children with sJIA may be included if the disease course suggests a persistent systemic inflammatory pattern, particularly for those with biologically active disease beyond the first year of treatment [24]. The evidence reviewed in earlier sections supports beginning surveillance at the diagnosis of the autoinflammatory syndrome rather than at first occurrence of proteinuria, given that subclinical injury precedes overt urinary findings by years [7,25,29]. Establishing a personal baseline for each biomarker at diagnosis appears valuable, since intra-patient trends over time may yield earlier signals than comparisons against population reference ranges alone [15,26].

### 8.2. What to Measure and How Often

Four monitoring elements emerge from the medical literature as offering complementary coverage of the injury *continuum* within the kidney [17,28,29,30,31]. Urinary NGAL, normalized to urinary creatinine, captures tubular stress earlier than glomerular markers shift [29,30]. Urinary albumin-to-creatinine ratio detects an emerging glomerular dysfunction and is best understood as a confirmatory rather than an early warning marker [6,27]. SAA quantification provides direct measurement of amyloidogenic substrate availability and reflects persistent inflammatory activity between attacks [17,26,40]. Renal ultrasonography with SWE, where available, provides a structural assessment of cortical stiffness that has been shown to correlate with subclinical disease activity [28,32,34]. In centres without access to quantitative SAA assays, hsCRP has been used as a surrogate, with the acknowledgement that this introduces some imprecision in patients with discordant SAA-CRP correlation [17].

Current available evidence suggests that surveillance frequency may benefit from stratification by syndrome-specific amyloid risk and current disease activity rather than uniform application across all patients [6,7,10]. Higher-risk patients, including those with TRAPS, M694V homozygous *MEFV* variants for FMF, or active CAPS disease despite optimized treatments, may warrant assessment at six-month intervals [10,13,14]. Standard-risk patients with stable and well-controlled disease may reasonably be assessed annually [6,27]. Active flare periods or the appearance of any biomarker abnormality should warrant intensified monitoring at three-month intervals until stability is re-established [17,29]. Such a tiered approach would concentrate surveillance intensity on the patients most likely to benefit from it.

### 8.3. Trigger Points for Biomarker-Guided Therapeutic Escalation in Amyloidosis

The clinical utility of this monitoring framework rests on the decision rules that govern action on biomarker findings. The evidence reviewed suggests that nephrology referral may be appropriately prompted by any of the following guideline-supported findings: new-onset microalbuminuria [6,26], SAA persistently elevated during intercritical periods despite optimized therapy [17,26], or the appearance of overt proteinuria, particularly if combined with hematuria, which warrants stricter evaluation for a correct individual differential diagnosis [35]. Urinary NGAL and renal SWE, by contrast, are not, on current evidence, independent clues for referral or escalation; persistent or rising values are better used to intensify monitoring and shorten reassessment intervals, prompting earlier repeat testing of the guideline-supported markers rather than immediate escalation [28,29,33,40].

A host of pediatric studies related to autoinflammatory syndromes has confirmed that early introduction of IL-1 blockade can produce more favorable renal outcomes when applied at the same disease stage [7,38]. This raises the question of whether biochemical resistance, demonstrated by persistent SAA elevation despite adherence to colchicine or IL-1 antagonists, might warrant weight alongside clinically defined treatment-resistance in driving therapeutic escalation. We emphasize that this remains a hypothesis: no prospective study has yet demonstrated that escalating therapy on the basis of biomarker abnormalities improves renal outcomes, and biomarker findings should not at present be treated as surrogate endpoints substituting for established clinical and histological criteria.

**Figure 4:**
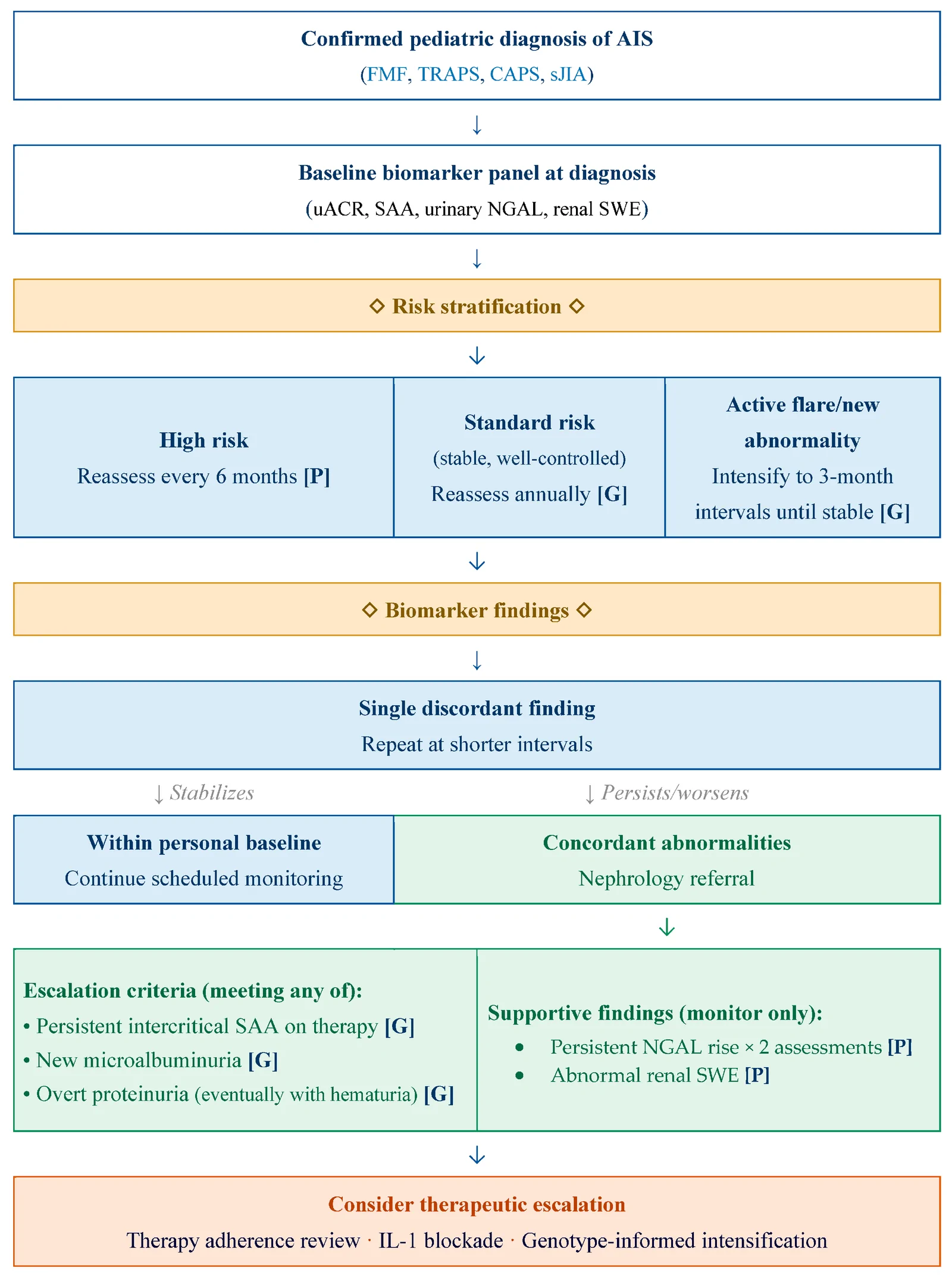
Proposed renal surveillance algorithm for pediatric autoinflammatory syndromes: baseline biomarker assessment at diagnosis is followed by risk-stratified monitoring intervals, with concordant abnormalities across modalities requiring nephrology referral and eventual consideration of therapeutic escalation (the dashed node indicates a biomarker-driven escalation criterion not yet incorporated into current guidelines). **[G]**: guideline-based (EULAR/PReS FMF recommendations); **[P]**: proposed. *Abbreviations:* AIS: autoinflammatory syndrome; CAPS: cryopyrin-associated periodic syndrome; FMF: familial Mediterranean fever; IL: interleukin; NGAL: neutrophil gelatinase-associated lipocalin; SAA: serum amyloid-A; sJIA: systemic juvenile idiopathic arthritis; SWE: shear wave elastography; TRAPS: tumor necrosis factor receptor-associated periodic syndrome; uACR: urinary albumin-to-creatinine ratio.

The current treatment paradigm in FMF reserves biological therapy for patients with persistent clinical attacks despite optimal dosing of colchicine, but the biomarker evidence summarized in this review suggests that a parallel biochemical criterion may merit consideration in future guidelines, particularly for patients carrying higher-risk genotypes [10,13,38]. The framework’s analytical value relies less in any single biomarker or threshold than in the concordance it can build across multiple modalities. Discordant findings can be re-evaluated at shorter intervals without immediate actions; concordant abnormalities across biochemical, structural, and clinical measures may provide stronger justification for therapeutic escalation than single-marker findings considered in isolation. Whether such an integrated approach can meaningfully shift pediatric autoinflammatory syndromes surveillance from late detection of established disease toward earlier characterization of accumulating risk is a question that prospective studies will need to address. Table 3 lists the evidence for markers useful in the renal surveillance framework in children with autoinflammatory syndromes, while Figure 4 proposes a renal surveillance algorithm in a synthetic fashion.

## 9. Discussion

Renal AA amyloidosis represents the most important issue of sustained and even subclinical inflammation in different autoinflammatory syndromes, capable of progressing silently to an irreversible renal failure. The argument advanced in this review rests on the alignment of three previously distinct strands of evidence that, when read together, support a fundamental shift in how pediatric autoinflammatory syndromes are monitored. The pathophysiological strand demonstrates that these conditions produce continuous rather than episodic inflammatory injury, with acute phase reactants often remaining elevated during the so-called intercritical periods: this occurs in a considerable proportion of clinically well patients, even when correctly treated with the proper treatments [17,26]. The biomarker strand demonstrates that subclinical renal injury is detectable years before frank and overt proteinuria appears, with urinary NGAL elevated in attack-free FMF children, emerging elastography data showing increased renal cortical stiffness in pediatric cohorts, and SAA persisting as an amyloidogenic stressor between flares [29,33,34]. The therapeutic strand demonstrates that IL-1 blockade halts amyloid progression and, at least in selected pediatric series, induces histological regression of established amyloid deposits [7,38]. Each of these findings, read separately, has limited prescriptive force. However, if read together, they may constitute the empirical foundation for reorienting pediatric autoinflammatory syndrome surveillance from a reactive proteinuria-based model toward proactive biomarker-guided risk stratification.

This reorientation should not be presented as settled science, and several reasonable counter-arguments deserve direct engagement. The most substantive is the absence of prospective randomized data demonstrating that biomarker-guided surveillance produces superior long-term renal outcomes compared with current practice. The evidence supporting earlier intervention is, in the main, cross-sectional, observational, and frequently single-centre [28,29,33,38]. Validated specific cut-off values for NGAL and SWE remain to be established, and the cost implications of expanded surveillance, particularly in resource-constrained settings, have not been formally analyzed. These limitations are genuine, and the framework outlined in this review should be understood as a novel hypothesis to test rather than a protocol to be adopted uncritically.

A further consideration concerns the potential harms of expanded surveillance itself. Because urinary NGAL and renal elastography currently lack disease- and age-specific thresholds, their use at scale carries a real risk of false-positive results, which in a screening context are not benign. They can prompt unnecessary repeat testing, imaging, or even renal biopsy, each with procedural and psychological cost in children; generate sustained anxiety in families already managing a chronic disease; and, most consequentially, drive treatment escalation to biologic therapy in children who would not have progressed, exposing them to the risks and costs of long-term immunomodulation without benefit. These are not hypothetical concerns but the predictable consequence of deploying markers without validated cut-offs, and they weigh directly against premature adoption. The economic dimension compounds this: the cost-effectiveness of adding quantitative SAA assays, serial urinary biomarkers, and specialized elastography to routine care has not been formally analyzed and would vary across health systems [17,29,33]. The approach proposed here should therefore be evaluated prospectively not only for whether it improves renal outcomes, but for whether its benefits outweigh the harms and costs of implementation.

A second consideration concerns the changing epidemiological landscape. In fact, the nutcracker syndrome data from contemporary pediatric FMF cohorts indicate that AA amyloidosis has become uncommon in adequately treated populations, with proteinuria now more often reflecting a structural vascular phenomenon than ongoing inflammatory damage [35].

One could reasonably argue, from this evidence alone, that the case for intensified surveillance is weaker than it might have been two decades ago. The counter-position, and the one this review takes, is that residual amyloidosis risk has become concentrated specifically in the patients most likely to be missed by current monitoring: those with intercritical biochemical activity despite apparent clinical control, those with non-FMF conditions for whom colchicine offers no equivalent protection, and those in geographic or socioeconomic settings where access to optimal therapies remains uneven [10,14,23,37]. The declining incidence of AA amyloidosis resulting from effective therapies does not diminish the need for surveillance; rather, it increases the importance of accurately identifying the smaller subgroup of patients who continue to carry a residual substantial risk. This requires surveillance tools with greater sensitivity than those designed for population-level screening.

A third consideration, and perhaps the most underappreciated, is the way current diagnostic reflexes lag behind contemporary therapeutic progress. The fact that nutcracker syndrome may be considered a relevant cause of proteinuria in young patients with colchicine-treated FMF, having been historically attributed to amyloidosis or to non-specific causes, illustrates how clinical thinking can fail to update at the pace of treatment success [35]. The same conceptual blind spot may operate in the opposite direction: subclinical amyloid injury risks being missed in a minority of patients because clinicians have correctly observed that overt amyloidosis has become rarer and have downgraded their suspicion accordingly.

Maintaining diagnostic vigilance in a successfully treated population is methodologically harder than maintaining it in an untreated one. The biomarker-guided framework proposed in this review is, in part, a response to that challenge, and the broader field would benefit from explicit recognition that surveillance protocols designed for an untreated disease era are not automatically appropriate for the contemporary age. The reframing of autoinflammatory syndromes as continuous rather than episodic diseases has implications that extend beyond the renal compartment. Different activity scoring systems have been developed for judging the clinical course with different organ involvement and treatment responses by using simple clinical or laboratory parameters [41,42]. Recent work on the development of an autoinflammatory disease damage index acknowledges precisely this conceptual territory, noting that organ damage can occur both before the initiation of therapy and during periods of ongoing subclinical inflammation [43].

The renal findings synthesized in this review can be read as one specific manifestation of a broader phenomenon affecting hepatic, cardiovascular, neurological, and pulmonary compartments, each with its own subclinical phase, its own emerging biomarkers, and its own potential for early intervention. The kidney is, in this sense, not unique but exemplary: it offers the clearest current pathway from continuous low-grade inflammation through detectable subclinical injury to a preventable long-term complication. Other organ systems may yet prove to follow similar trajectories, and the analytical approach developed for renal surveillance may be generalizable in ways that this review does not attempt to specify.

Finally, the synthesis offered herein should be situated within the broader argument that pediatric autoinflammatory syndromes are systemic diseases whose ultimate impact extends well beyond fever phenotype. The recurrent fever that brings these children to clinical attention is, in the most consequential sense, a presenting feature. What lies beneath that presenting feature is a process of continuous immunological dysregulation whose effects accumulate silently across the developmental and adolescent years, and whose long-term consequences declare themselves at the organ level only after a window for prevention has often passed. Subclinical inflammation may work as a smoldering fire even in children, and our task as clinicians managing pediatric autoinflammatory syndromes requires looking for the visible markers of disease activity toward the slower processes operating underneath. Successfully making this conceptual shift hinges on prioritizing the integration of existing evidence into routine pediatric care rather than waiting for new data.

## 10. Gaps in the Evidence and Future Directions

The main evidence gap underpinning this review is methodological. Large prospective pediatric cohorts tracking subclinical renal biomarkers from the diagnosis of autoinflammatory syndromes through long-term follow-up do not yet exist in the volume or design required to establish definitive predictive thresholds [17,29,43]. International collaborative registries such as Eurofever or AIDA (Autoinflammatory Disease Alliance) provide an established infrastructure for multi-centre data collection, but systematic integration of urinary NGAL, SAA, and renal elastography into these platforms has been incomplete [7,27]. Prospective studies linking individual biomarker trajectories to subsequent renal outcomes over five- to ten-year follow-up periods would generate the best evidence base required to transform the framework proposed here from a literature-derived synthesis into a clinical protocol.

A second priority is the development of autoinflammatory syndrome-specific reference ranges. Most available NGAL and SWE data in child cohorts derive from cross-sectional comparisons against healthy controls rather than from prospective studies tracking which threshold values predict subsequent amyloid progression [29,30,33,43]. Standardization of acquisition protocols for renal elastography across centres remains a precondition for meaningful cross-cohort comparison, and quantitative SAA assays require validated pediatric reference ranges before they can support uniform interpretation. These are achievable goals that would benefit from coordinated effort across the major pediatric rheumatology research networks.

A third opportunity concerns therapeutic trial design. Randomized data comparing early biomarker-triggered escalation to IL-1 blockade against standard symptom-driven escalation in colchicine-treated FMF patients with persistent biochemical activity or in TRAPS and CAPS refractory patients would directly test the central proposition advanced by this review [7,21,38]. Furthermore, because current claims regarding the reversibility of established amyloid deposits rely largely on small observational series, such a purpose faces real challenges of recruitment, ethical complexity in the pediatric population, and the need for sufficiently longer follow-up to capture meaningful renal endpoints. These challenges are surmountable through multi-centre collaboration, and a robust comparative trial of this design should represent the logical next step in translating the accumulating biomarker evidence into validated clinical practice. Until such evidence becomes available, the framework outlined here should be applied with the explicit understanding that it synthesizes current best evidence rather than embodying definitive proof.

## 11. Conclusive Remarks

Renal AA amyloidosis in pediatric autoinflammatory syndromes should be regarded as a substantially mitigable complication rather than an inevitable endpoint. Although early suppression of systemic inflammation can prevent it under optimal conditions, a residual risk persists in practice through high-risk genotypes such as *MEFV* M694V homozygosity, delayed diagnosis, partial therapeutic resistance, adherence difficulties, and unequal global access to biologic therapies. During the multi-year interval between disease onset and overt nephropathy, the kidney undergoes subclinical injury in which targeted intervention can stabilize renal function and potentially avert irreversible structural failure. The continued reliance on overt proteinuria as the sole threshold for nephrology referral is becoming harder to justify, particularly given the demonstrated capacity of IL-1 blockade to halt and, in selected cases, reverse amyloid progression. The integrated biomarker approach reviewed here, combining microalbuminuria, urinary NGAL, SAA, and renal SWE, offers a clinically tractable pathway toward earlier identification of at-risk children and toward more rational decisions about therapeutic escalation [44].

Before it can enter routine care, this approach requires prospective validation, the development of syndrome-specific reference ranges, and adaptation to local resource environments, together with a careful weighing of its benefits against the harms and costs of surveillance at scale. The direction, however, is clearer than the unresolved science might suggest: surveillance of the pediatric kidney in autoinflammatory syndromes should shift from the late detection of established disease toward the early characterization of accumulating risk. The candidate tools to make that shift already exist, confirming their value. Then, using them where they can still change a child’s long-term outcome is the task now before the field.

## Figures and Tables

**Table 1:** Study-level evidence for candidate biomarkers of early renal involvement in pediatric autoinflammatory syndromes, with microalbuminuria as the current comparator (no study has prospectively tested urinary NGAL or renal SWE against an incident-amyloidosis endpoint; most available data derive from cross-sectional comparisons of patients with familial Mediterranean fever with healthy controls).

*Biomarker (Study)*	*Population*	*Number*	*Design*	*Endpoint Assessed*	*Sensitivity for* *Amyloidosis*	*Follow-Up*	*Evidence Level*
Urinary NGAL [29]	Attack-free FMF children *versus* healthy controls	40/38	Cross-sectional, case–control	NGAL higher *versus* controls; correlated with attacks/year	Not assessed	None (single time-point)	Limited/preliminary
Urinary NGAL [31]	Narrative review of NGAL in FMF	N/A	Narrative review	Biological plausibility as tubular marker	Not assessed	Not assessed	Supporting rationale only
Renal SWE [28]	FMF, FMF- amyloidosis subgroup, and controls	27/11/38	Prospective, case–control	Renal stiffness higher *versus* controls; did not separate amyloid from non-amyloid kidneys	Not assessed as a predictive test	None (single time-point)	Limited/preliminary
Renal SWE [33]	Pediatric FMF *versus* controls	50	Cross-sectional	SWE higher *versus* controls; no correlation with disease activity scores	Not assessed	None (single time-point)	Limited/preliminary
Renal SWE [34]	Children with FMF *versus* controls	79/79	Prospective, cross-sectional	Renal SWE *versus* controls (adjunctive marker of renal involvement)	Not assessed	None (single time-point)	Limited/preliminary
SAA [17]	Attack-free FMF patients	183	Cross-sectional	Intercritical SAA and inflammatory monitoring	Associated with amyloidogenic risk	None	Moderate (guideline-referenced)
Microalbuminuria/uACR	FMF surveillance populations	Multiple	Guideline-adopted practice	Established glomerular marker	Confirmatory, post-deposition	Variable	High (guideline- referenced)

*Abbreviations:* FMF, familial Mediterranean fever; NGAL, neutrophil gelatinase-associated lipocalin; SAA, serum amyloid-A; SWE, shear wave elastography; uACR, urinary albumin-to-creatinine ratio.

**Table 2:** Principal pharmacological therapies relevant to renal AA amyloidosis in pediatric autoinflammatory syndromes, with each agent’s mechanism, principal indication, and current evidence for an effect on renal amyloidosis. Colchicine is first-line prophylaxis in FMF; IL-1 and IL-6 blockade are reserved for colchicine-resistant FMF and for syndromes in which colchicine is ineffective. Evidence for regression of established amyloidosis derives largely from small pediatric series and is strongest when treatment begins before irreversible glomerular damage.

*Agent*	*Mechanism*	*Principal Indication*	*Evidence on Renal Amyloidosis*
Colchicine	Stabilizes the pyrin inflammasome	FMF (first-line prophylaxis)	Prevents amyloidosis and lowers its incidence to near zero in adherent cohorts; ineffective in TRAPS, CAPS, and sJIA; 5–10% of FMF patients show partial or complete resistance
Anakinra	Recombinant IL-1 receptor antagonist	Colchicine-resistant FMF; TRAPS; CAPS	Halts amyloid progression; partial remission of amyloidosis-related nephrotic syndrome reported in FMF
Canakinumab	Long-acting anti-IL-1β monoclonal antibody	Colchicine-resistant FMF; TRAPS; CAPS; sJIA	Reduced proteinuria and stabilized renal function in FMF-related amyloidosis, with proteinuria abolished in a subset of treated patients
Tocilizumab	Anti-IL-6 receptor monoclonal antibody	sJIA; selected refractory cases of AA amyloidosis	Reduced serum amyloid-A and stabilized renal function in selected cases; most relevant where IL-6 predominates (as in sJIA)

*Abbreviations:* CAPS, cryopyrin-associated periodic syndrome; FMF, familial Mediterranean fever; IL, interleukin; sJIA, systemic juvenile idiopathic arthritis; TRAPS, tumor necrosis factor receptor-associated periodic syndrome.

**Table 3:** Strength-of-evidence appraisal of the components of the proposed renal surveillance framework for pediatric autoinflammatory syndromes. Each element is classified by its supporting evidence base, the qualitative strength of that evidence, and its current status as either guideline-based or proposed. Guideline-based components derive from EULAR/PReS recommendations for the management of FMF; the proposed components rest on preliminary data and are advanced as hypotheses for prospective validation (this appraisal is qualitative and does not constitute a formal GRADE assessment).

*Surveillance Element*	*Evidence Base*	*Strength*	*Status*
uACR/microalbuminuria monitoring	EULAR/PReS FMF recommendations (2016; 2024 update)	Moderate	Guideline-based
Intercritical SAA monitoring	EULAR/PReS 2024 update (preferential SAA and proteinuria monitoring); FMF cohort data	Moderate	Guideline-endorsed
Risk-stratified monitoring intervals	EULAR/PReS 2024 update (3–6-monthly, more frequent if uncontrolled); observational cohort studies on high-risk genotypes	Moderate	Guideline-based
Urinary NGAL	Small, single-centre, cross-sectional pediatric FMF studies; no validated cut-offs	Limited/ preliminary	Proposed
Renal shear wave elastography (SWE)	Small pediatric FMF studies; no standardized protocols or reference ranges	Limited/ preliminary	Proposed
Biomarker-triggered therapeutic escalation	Extrapolated from IL-1 blockade outcome data; not prospectively tested	Limited/ preliminary	Proposed

*Abbreviations:* EULAR: European Alliance of Associations for Rheumatology; FMF: familial Mediterranean fever; IL-1: interleukin-1; NGAL: neutrophil gelatinase-associated lipocalin; PReS: Paediatric Rheumatology European Society; SAA: serum amyloid-A; SWE: shear wave elastography; uACR: urinary albumin-to-creatinine ratio.

## Data Availability

No new data were created or analyzed in this study. Data sharing is not applicable to this article.

## Abbreviations

The following abbreviations are used in this manuscript:

IL	interleukin
NGAL	neutrophil gelatinase-associated lipocalin
FMF	familial Mediterranean fever
SAA	serum amyloid-A
CRP	C-reactive protein
hsCRP	high-sensitivity C-reactive protein
TRAPS	tumor necrosis factor receptor-associated periodic syndrome
CAPS	cryopyrin-associated periodic syndrome
NOMID	neonatal-onset multisystem inflammatory disease
sJIA	systemic juvenile idiopathic arthritis
AIS	autoinflammatory syndrome
CKD	chronic kidney disease
ESRD	end-stage renal disease
uACR	urinary albumin-to-creatinine ratio
SWE	shear wave elastography
